# Supplementary material for: In vivo spatiotemporal control of voltage-gated ion channels by using photoactivatable peptidic toxins
Source: Nat Commun. 2022 Jan 20;13:417. doi: 10.1038/s41467-022-27974-w (PMC8776733; doi:10.1038/s41467-022-27974-w)
Supplement: Supplementary file 1 — Supplementary Information [file 41467_2022_27974_MOESM1_ESM.pdf]

In vivo spatiotemporal control of voltage-gated ion channels by using photoactivatable peptidic toxins

Jérôme Montnach[1,2], Laila Ananda Blömer[2,3], Ludivine Lopez[1,2,4], Luiza Filipis[2,3], Hervé Meudal[5], Aude Lafoux[6], Sébastien Nicolas[1,2], Duong Chu[7], Cécile Caumes[4], Rémy Béroud[4], Chris Jopling[8], Frank Bosmans[9], Corinne Huchet[6], Céline Landon[5], Marco Canepari[2,3], Michel De Waard[1,2,4,*]

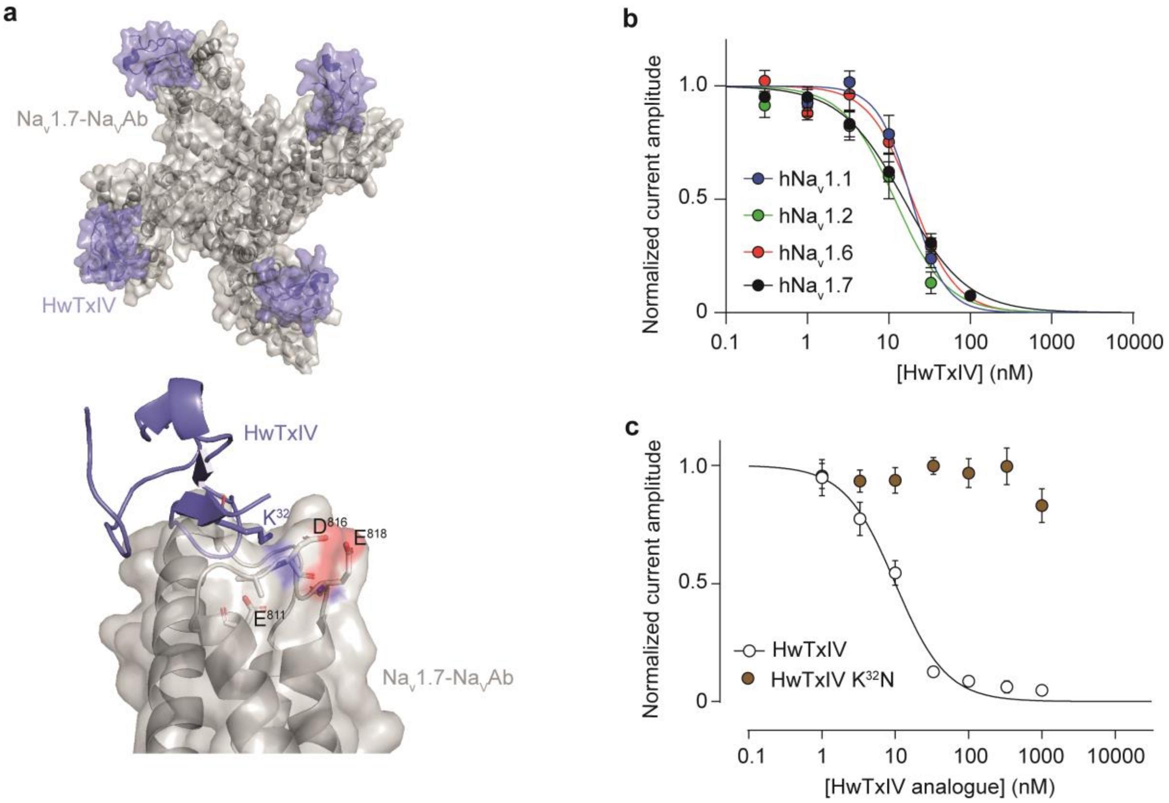

Supplementary Figure 1. (a) Molecular structure of the HwTxIV / NaV1.7-NavAb chimera complex (PDB code 7K48). (b) Average dose-response curves (mean ± SEM) for inhibition of hNav1.1 (blue), hNav1.2 (orange), hNav1.6 (red) and hNav1.7 (black) currents by non-caged HwTxIV. The data were fitted according to a Hill equation study (hNaV1.1: 18.9 ± 1.2 nM, n=29; hNaV1.2: 11.9 ± 1.1 nM, n=38; hNaV1.6: 19.5 ± 1.3 nM, n=55; hNaV1.7: 15.2 ± 1.1 nM, n=81). (c) Average dose-response curves for hNav1.7 current inhibition by the nHwTxIV (black) and nHwTxIV-K32N mutant (brown). The data were fitted according to a Hill equation (IC50 > 1 μM, n=44 for nHwTxIV-N32 versus IC50 = 9.9 nM ± 0.9 nM, n=45 for nHwTxIV).

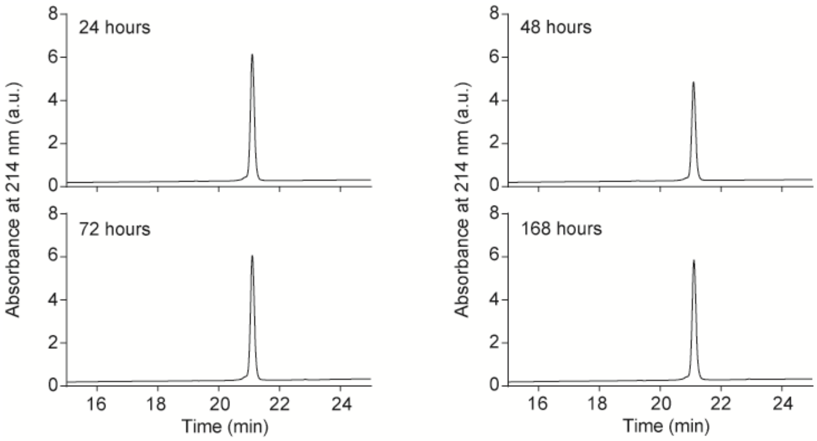

Supplementary Figure 2. Analytical RP-HPLC profiles of HwTxIV-Nvoc after different times in the dark demonstrating perfect stability of the caged peptide.

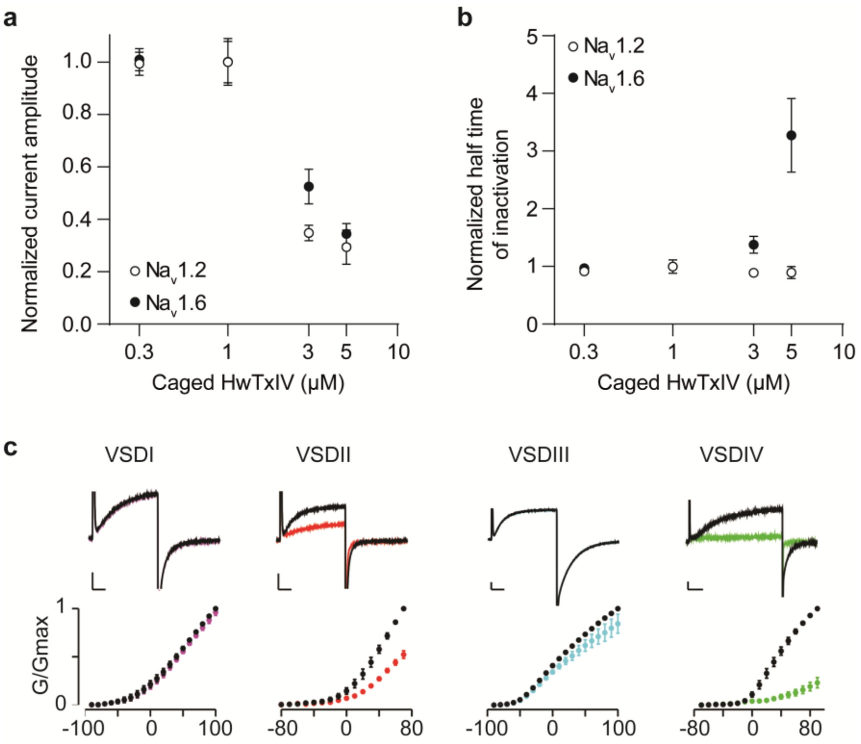

Supplementary Figure 3. (a) Average dose-response curves for hNa$_V$1.2 (black circles, n=11) and hNa$_V$1.6 (open circles, n=12) peak current inhibition by high concentrations of HwTxIV-Nvoc. (b) Average dose-response curves for hNa$_V$1.2 (black circles, n=11) and hNa$_V$1.6 (open circles, n=12) half-time of inactivation by high concentrations of HwTxIV-Nvoc. (c) Top: K^+ currents from hNa$_V$1.6/rK$_V$2.1 chimeras harboring the VS domains of hNa$_v$1.6. Currents were elicited by depolarizations to +40 mV. Currents are shown before (black) and in the presence (colored traces) of 5 μM caged HwTxIV. Colors denote the domain from which the hNa$_V$1.6 paddle motif was transferred: VSDI in magenta; VSDII in red; VSDIII in cyan; VSDIV in green. Scale bars: 20 ms (abscissa) for VSDI-III, 40 ms for VSDIV; and 0.5 μA (ordinate axis). Bottom: Tail current conductance-activation (G-V) relationships for each of the hNa$_V$1.6/rK$_V$2.1 chimeras (n = 4). The holding voltage was -90 mV and the tail voltage was -70 mV (or -90 mV for VSDIII).

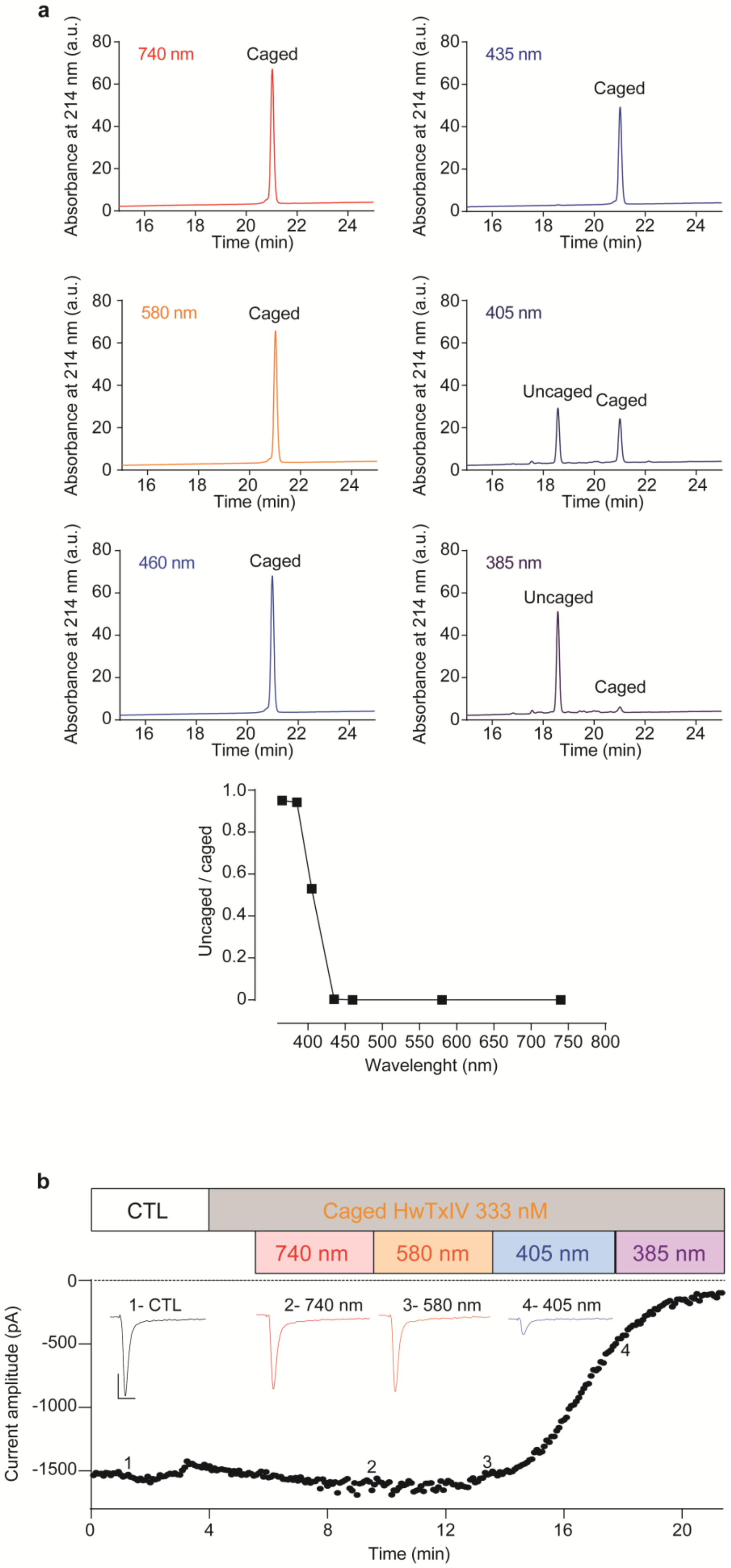
a
740 nm
Caged
435 nm
Caged
580 nm
Caged
405 nm
Uncaged
Caged
460 nm
Caged
385 nm
Uncaged
Caged
Absorbance at 214 nm (a.u.)
Time (min)
Uncaged / caged
Wavelenght (nm)
b
CTL
Caged HwTxIV 333 nM
740 nm
580 nm
405 nm
385 nm
1- CTL
2- 740 nm
3- 580 nm
4- 405 nm
Current amplitude (pA)
Time (min)

Supplementary Figure 4. (a) Top: Analytical RP-HPLC profiles of photolysis of HwTxIV-Nvoc at different wavelengths (10 min, power > 18 mW/cm^2). Bottom: Uncaged/Caged ratio of RP-HPLC chromatogram peak area at different wavelengths. (b) Representative current traces and time course of hNa$_V$1.6 current inhibition by HwTxIV-Nvoc illuminated at different wavelengths. Scales: 2 ms, 500 pA.

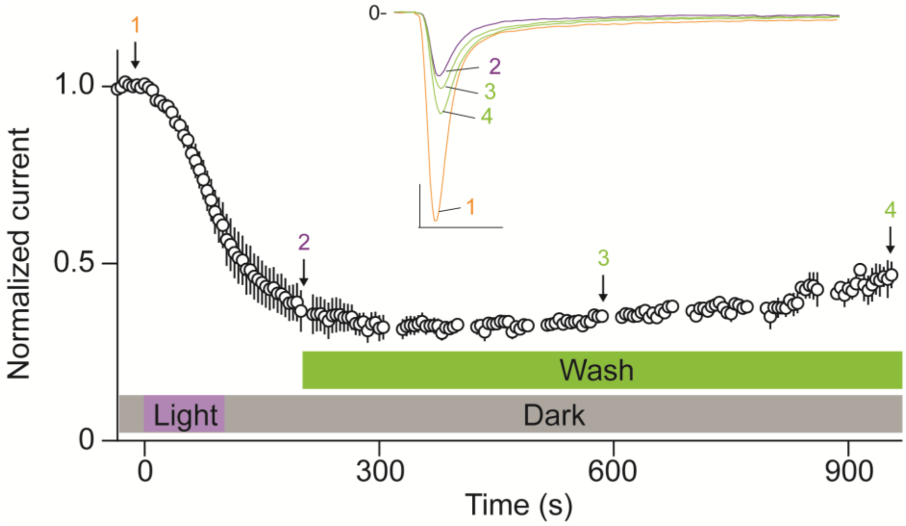

Supplementary Figure 5. Reversibility of $hNa_V1.6$ inhibition after HwTxIV-Nvoc uncaging. Representative current traces (inset) and average normalized time course of $hNa_V1.6$ current inhibition by HwTxIV-Nvoc illuminated at 365 nm 45 mW/cm². Wash of uncaged HwTxIV induces a partial 16% current recovery after 11 min of wash (n=6, mean ± SEM). Scales: 2 ms, 1 nA.

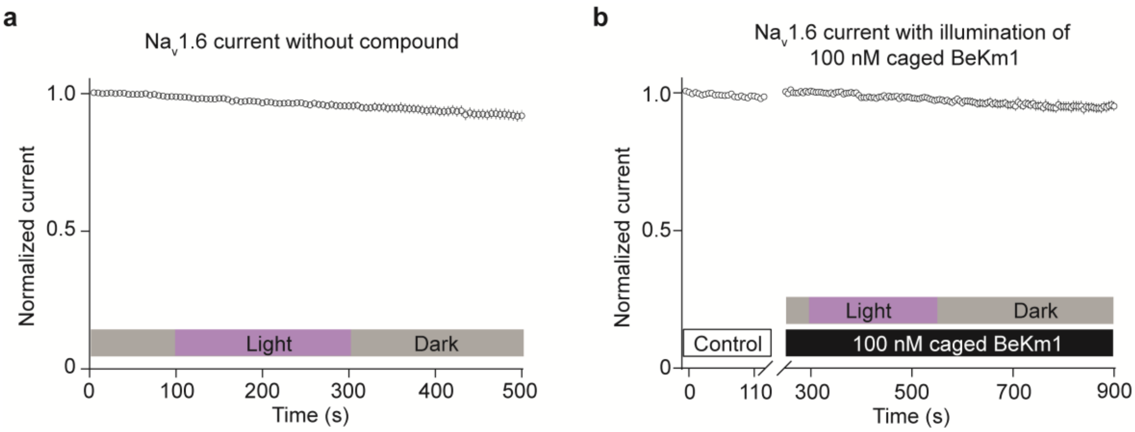

Supplementary Figure 6. (a) Average normalized time courses of $hNa_V1.6$ peak current inhibition by illumination (4 min, 365 nm, 45 mW/cm^2) without any compound (n=22, mean ± SEM). (b) Average normalized time courses of $hNa_V1.6$ peak current inhibition by illumination (4 min, 365 nm, 45 mW/cm^2) of 100 nM BeKm1-Nvoc (n=14, mean ± SEM). BeKm1 has no reported inhibitory effects on $hNa_V1.6$ peak current inhibition.

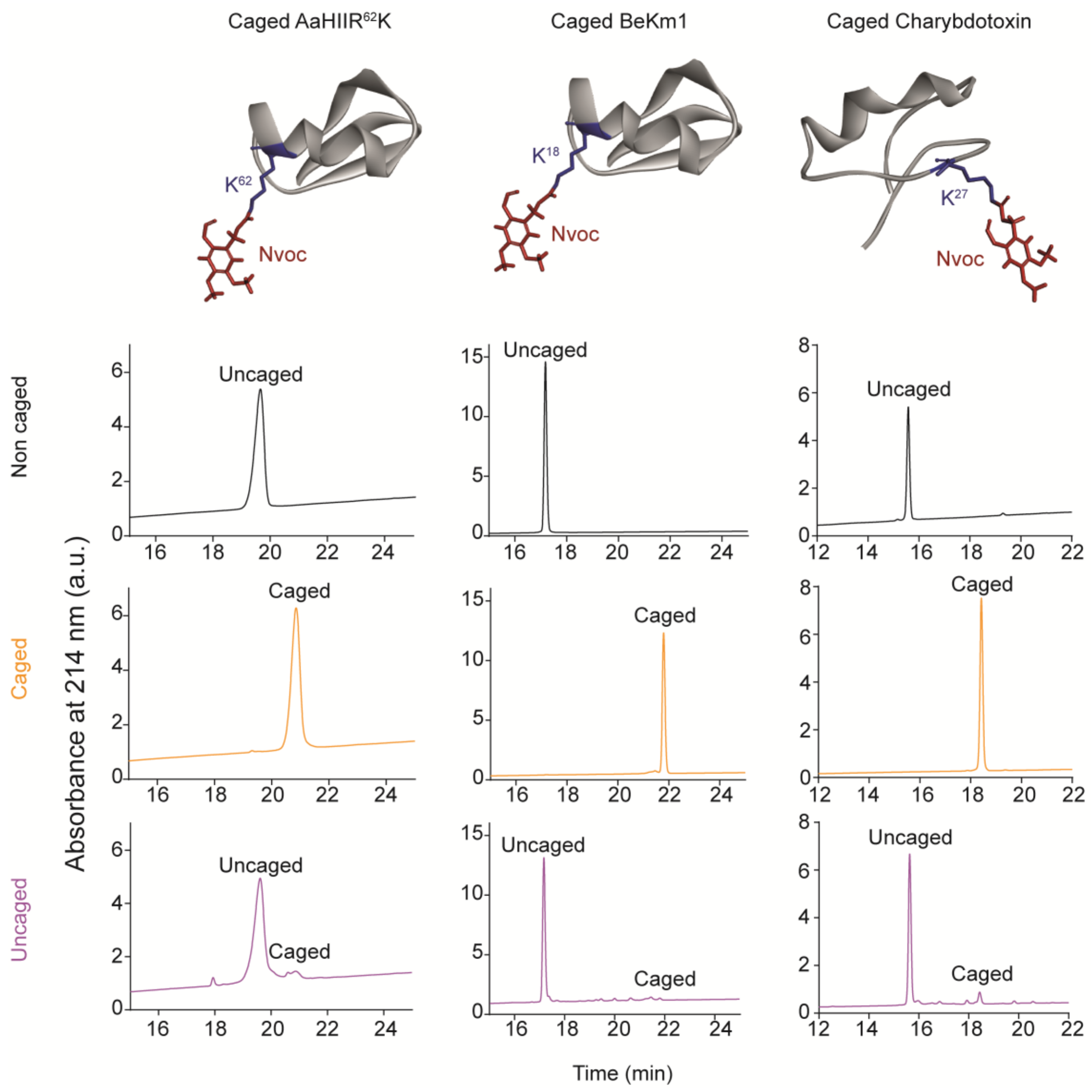

Supplementary Figure 7. Top: 3D structure of other Nvoc-protected toxins. Bottom: Analytical RP-HPLC profiles of non-caged (black), caged (orange) and uncaged (purple) AaHII-R^{62}K (left, PDB code 6NT4), BeKm1 (middle, PDB code 1J5J) and charybdotoxin (right, PDB code 4JTA).

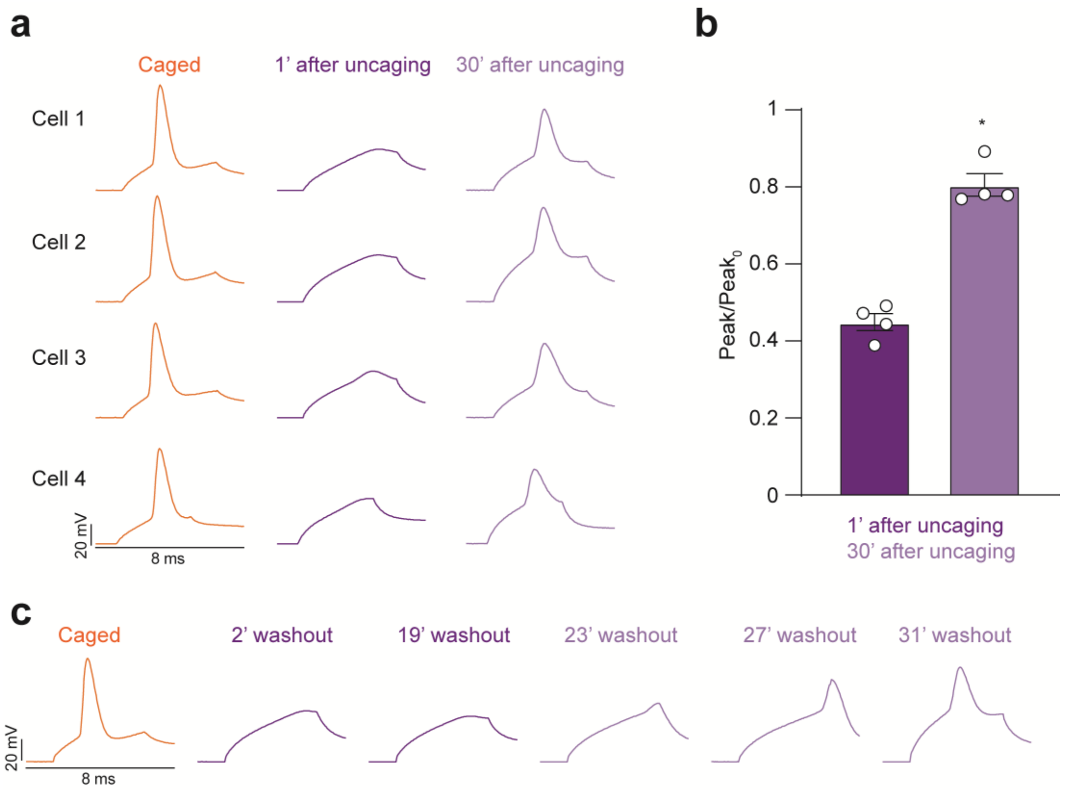

Supplementary Figure 8. HwTxIV reversibility in L5 pyramidal neurons from mouse brain slices. (a) APs in the presence of 2.5 µM HwTxIV-Nvoc (orange traces), and 1 and 30 minutes after uncaging the toxin for 4 different cells. (b) mean ± SEM (n = 4 cells) of the depolarization peak, normalized to the caged values, 1 minute (0.45 ± 0.02) and 30 minutes after photolysis (0.81 ± 0.03). "*" indicates a significant increase of the peak ($p < 0.05$, paired t-test). (c) Example of kinetics of recovery of AP amplitude after photolysis of HwTxIV-Nvoc.

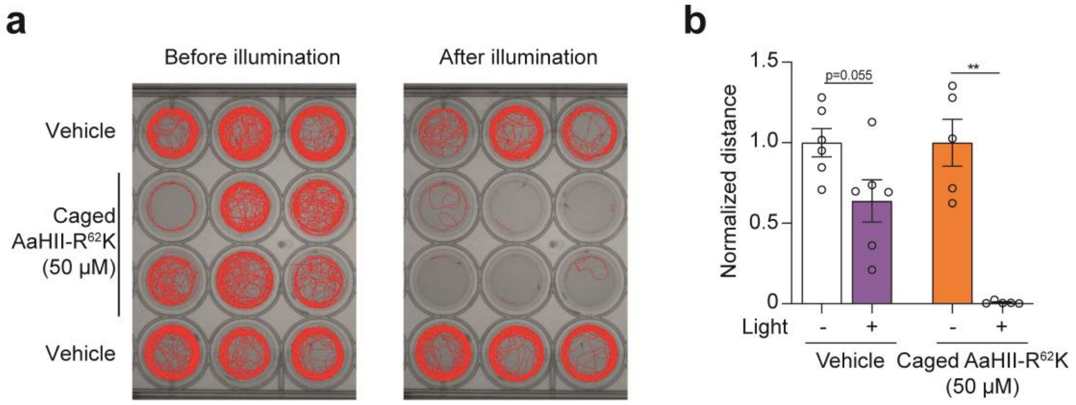

Supplementary Figure 9. In vivo photocontrol of caged AaHII-R^{62}K induces paralysis of zebrafish larvaes. (a) Representative traces of zebrafish larvaes movements in larvaes injected with vehicle (top and bottom) or AaHII-R^{62}K-Nvoc (50 µM) prior or after 365 nm illumination. (b) Normalized distance traveled by larvaes prior or after illumination after vehicle or AaHII-R^{62}K-Nvoc injection. (n=6 in each group, mean ± SEM; ** $p<0.01$ versus vehicle, paired t-test).

Supplementary Table 1. IC_{50} values of native HwTxIV (nHwTxIV) and HwTxIVG^{1}G^{4}K^{36} (HwTxIV) on hNa$_V$1.1, hNa$_V$1.2, hNa$_V$1.6 and hNa$_V$1.7 ion channels.

	nHwTxIV (nM)	HwTxIVG^{1}G^{4}K^{36} (nM)
hNa$_V$1.1	50.7 ± 1.2	18.9 ± 1.2
hNa$_V$1.2	8.3 ± 1.2	11.9 ± 1.1
hNa$_V$1.6	154.3 ± 1.2	19.5 ± 1.3
hNa$_V$1.7	9.9 ± 0.9	15.2 ± 1.1

Supplementary Data 1. Table of ^{1}H NMR chemical shifts of the non-caged HwTxIV.
