# Supplementary material for: In vivo spatiotemporal control of voltage-gated ion channels by using photoactivatable peptidic toxins
Source: Nat Commun. 2022 Jan 20;13:417. doi: 10.1038/s41467-022-27974-w (PMC8776733; doi:10.1038/s41467-022-27974-w)
Supplement: Supplementary file 3 — Description of Additional Supplementary Files [file 41467_2022_27974_MOESM3_ESM.pdf]

Title: Supplementary Movie 1.
Description: Animation illustrating the block of sodium influx after HwTxIV-Nvoc illumination. The movie is divided in three episodes. The first episode shows the Na^+ influx in the AIS associated with the generation of an AP using a false color scale and at fast temporal resolution. The second episode shows, at slower temporal resolution, the UV flash uncaging HwTxIV-Nvoc over the soma and the AIS. The third episode shows the absence of Na^+ influx in the AIS after uncaging HwTxIV-Nvoc even when the somatic membrane potential is depolarized to the same level of the AP peak before uncaging HwTxIV-Nvoc.

Title: Supplementary Movie 2.
Description: Video recorded using Ethovision setup of zebrafish larvae injected with 50 μM AaHII-R^{62}K-Nvoc prior to illumination.

Title: Supplementary Movie 3.
Description: Video recorded using Ethovision setup of zebrafish larvae injected with 50 μM AaHII-R^{62}K-Nvoc after 365 nm illumination.

Title: Supplementary Data 1.

Description: Table of ^{1}H NMR chemical shifts of the non-caged HwTxIV.
